# 37-year-old Transgender Man with Fevers, Dysuria, and Sudden Decompensation

**DOI:** 10.5811/cpcem.2020.4.45956

**Published:** 2020-07-20

**Authors:** Emily R. Fleming, J. David Gatz

**Affiliations:** * University of Wisconsin University Hospital, BerbeeWalsh Department of Emergency Medicine, Madison, Wisconsin; † University of Maryland School of Medicine, Department of Emergency Medicine, Baltimore, Maryland

**Keywords:** Clinicopathological cases, infectious disease, drug reaction

## Abstract

**Introduction:**

Patients in the emergency department may experience sudden decompensation despite initially appearing stable.

**Case Presentation:**

A 37-year-old transgender man presented to the emergency department (ED) with several months of fevers, myalgias, and weight loss. The patient acutely became febrile, tachycardic, and hypotensive after an initially reassuring assessment in the ED.

**Discussion:**

This case takes the reader through the differential diagnosis and work-up of the decompensating patient initially presenting with subacute symptoms.

## CASE PRESENTATION (Dr. Emily Fleming)

A 37-year-old transgender man presented to the emergency department (ED) of an urban, academic medical center in May with a four-month long course of symptoms including intermittent fevers, dysuria, and generalized malaise. He reported seeing his primary care provider (PCP) in January for dysuria and malaise after unprotected sex, tested positive for chlamydia, and was treated with azithromycin. He presented to his PCP again in March for what was now two months of weight loss, dysuria, fatigue, and body aches. Laboratory testing at that time included a complete blood count, basic metabolic panel, and thyroid stimulating hormone that were all within normal limits. A mononucleosis spot test and human immunodeficiency virus (HIV) test were both negative, and a urinalysis at that time was unrevealing.

In the ED the patient reported continued weakness, headaches, weight loss, and whole-body aches. He also reported intermittent fevers over the prior two weeks, measured at home as high as 101° Fahrenheit (F), coupled with 5–10 days of dysuria and suprapubic discomfort without any vaginal or rectal discharge. He received moderate relief from acetaminophen and ibuprofen at home.

He reported a history of anxiety, depression, type 2 diabetes, hyperlipidemia, polycystic ovarian syndrome, asthma, and prior pulmonary embolism (PE). His prior surgeries included cholecystectomy, hysterectomy, and double mastectomy. He was taking sertraline, trazadone, amitriptyline, metformin, and testosterone. He denied any tobacco, alcohol, or illicit drug use. He reported being sexually active with male partners and not routinely using barrier protection. He reported an allergy to hydrocodone-acetaminophen.

Vital signs were as follows: temperature 98.5°F, blood pressure 125/76 millimeters of mercury (mmHg), pulse 69 beats per minute (bpm), respiratory rate 16 breaths per minute, oxygen saturation 99% on room air, and a body mass index of 28.2 kilograms per meter squared. Physical exam revealed an anxious but non-toxic man. Head was normal in appearance and atraumatic. Conjunctivae were normal. Mucus membranes were slightly dry. Neck was supple, with no pain on flexion, and no lymphadenopathy. The heart rate was normal, and the rhythm was regular. There was no murmur. Lungs were clear to auscultation bilaterally and breathing was unlabored. The chest wall was notable for post-mastectomy scars. The abdomen was soft and nondistended, but with suprapubic tenderness to palpation. The extremities were warm and well perfused, and not edematous. Cranial nerves II–XII were intact, and gait and strength assessments were unremarkable. Skin was normal in appearance, without any lesions or rashes. Vaginal exam revealed a surgically absent cervix, and no bleeding, discharge, or adnexal tenderness. There were no hemorrhoids or other fluctuant mass palpable on rectal examination, but the patient did report moderate discomfort.

**Image 1:**
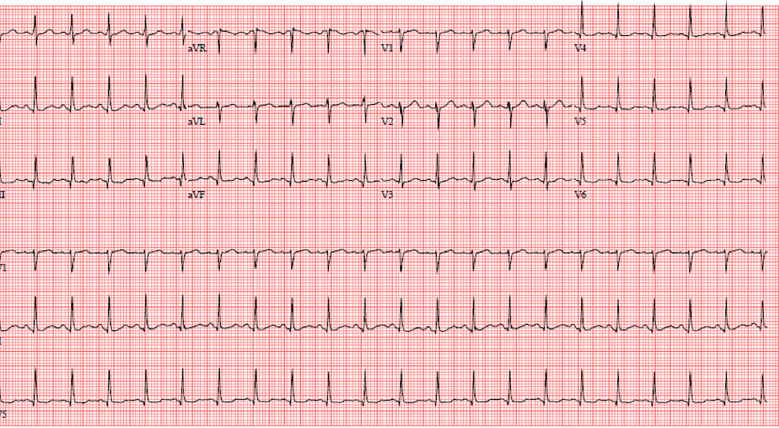
Electrocardiogram of a 37-year-old male with sudden decompensation, taken while in the emergency department.

**Image 2:**
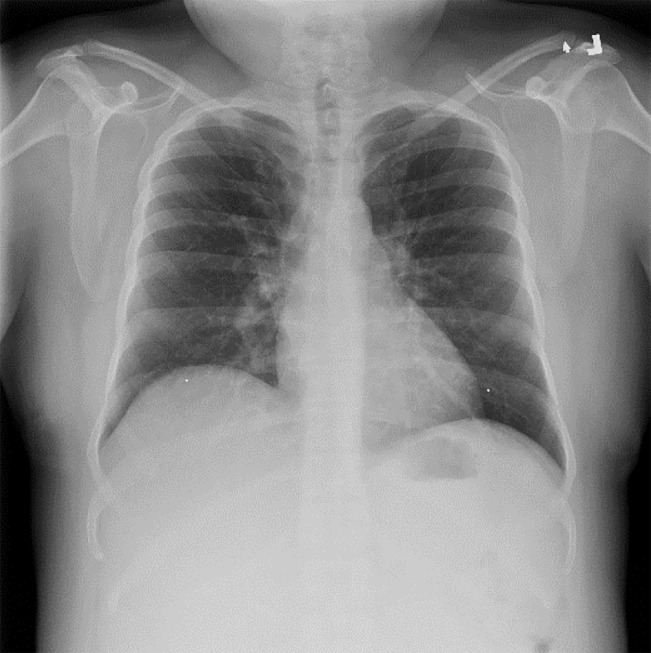
Anterior-posterior chest radiograph of a 37-year-old transgender male with sudden decompensation while in the emergency department.

The patient’s laboratory results, summarized in Table 1, did not require immediate intervention. He was treated in the ED with intravenous (IV) fluids, a nonsteroidal anti-inflammatory medication (NSAID), and was empirically treated with oral azithromycin and intramuscular (IM) ceftriaxone. Approximately one to two hours later a nurse went to discharge the patient but found his vitals to be grossly abnormal, including a temperature of 102.6°F, blood pressure 90/50 mmHg, pulse 135 bpm, respiratory rate 20 breaths per minute, and an oxygen saturation of 99% on room air. He now reported significant 8/10 full-body aches and severe headache. On examination he was now diaphoretic, rigoring, and tachycardic. His lungs remained clear to auscultation, while his abdomen remained soft and nondistended but still tender to palpation over the suprapubic region. Blood cultures and a lactate were sent because of this acute change. The patient additionally had an electrocardiogram (Image 1) and chest radiograph (Image 2). An additional test was sent from the ED, which confirmed the diagnosis.

## FACULTY DISCUSSION (Dr. J. David Gatz): A TALE OF TWO PATIENTS

What a case! And what a story! I cannot help but look across my desk to the bookshelf housing my haphazard collection of classic literature mixed between emergency medicine texts. I have always believed that each patient has a story to tell. While some stories are action and others tragedy, many stories from the ED are mystery. We are presented in this case with a puzzling “pan-positive” review of systems and a small novel of additional information; it’s overwhelming to know where to begin! Faced with such a task, I have elected to channel my own inner Charles Dickens and map out this patient’s story.

**CPC-EM Capsule**

What do we already know about this clinical entity?

The Jarisch-Herxheimer reaction is commonly encountered when treating syphilis with a penicillin and can cause significant changes in a patient’s vital signs.

What makes this presentation of disease reportable?

This case describes an unexpected Jarisch-Herxheimer reaction while empirically treating a patient for gonorrhea with ceftriaxone.

What is the major learning point?

The Jarisch-Herxheimer reaction can occur while treating spirochetes other than syphilis, and while using antibiotics other than penicillin.

How might this improve emergency medicine practice?

Clinicians should anticipate this possible reaction given the high prevalence of syphilis and common use of empiric antibiotics for sexually transmitted infections.

The scene is set with a 37-year-old trans-male patient with an assortment of common chronic medical conditions. His medications appropriately match these diagnoses. The patient’s testosterone supplementation and prior surgical procedures are also consistent with his gender transition. Foreshadowing or red herring, the patient has an otherwise unexplained history of PE. I must assume it was provoked from a prior surgery and successfully treated given that the patient is not on any chronic anticoagulation.

The story begins to build as we learn about unprotected receptive intercourse requiring prior treatment of chlamydia. There were otherwise no unique exposures. A couple months later our protagonist experienced dysuria and numerous systemic symptoms including weight loss and myalgias despite grossly negative laboratory studies. Fast forward two more months and these symptoms were joined by headaches, fevers, chills, and suprapubic discomfort. A fresh set of laboratory studies were, once again, grossly normal.

The patient was empirically treated with NSAIDs and typical empiric antibiotics for sexually transmitted infections, at which point we as readers suddenly experience a major plot twist! One of my favorite plotlines is in Dickens’ *A Tale of Two Cities*, which features several characters traveling back and forth to Paris and London in the late 1700s. These cities are in stark contrast to one another. While London prospers, Paris descends into the chaos preceding the French Revolution. We witness a similar stark contrast in the story of our patient – who suddenly went from relatively stable to hypotensive, tachycardic, tachypneic, and febrile.

But what was the source of this sudden decompensation? And how did it tie into our protagonist’s backstory? After careful deliberation I have narrowed it to four major possibilities. First, you will recall, our patient has a history of otherwise unexplained venous thromboembolism. An acute PE, especially if massive, could cause tachycardia, tachypnea, hypotension, and even fever!^1^ But this fails to explain any of the subacute symptoms that led our patient to present in the first place. An underlying oncologic process could have predisposed the patient to PE and caused generalized malaise, but there is nothing specific to support such a diagnosis. Similarly, an ingestion could have caused a sudden change in vital signs. A sympathomimetic causes tachycardia, tachypnea, and an increased body temperature, but typically induces *hyper*-tension instead of *hypo*-tension. Similarly, an anticholinergic ingestion could cause tachycardia and an increase in body temperature. But we are not given any indication or history of such exposures and, once again, this fails to explain the preceding subacute course of symptoms.

This leaves us looking for more clues from the patient’s exam. He noted discomfort during the vaginal and rectal exam. Is it possible the provider was palpating a tender abscess? The indolent growth of an abscess could explain many of the patient’s chronic symptoms, and a sudden rupture from palpation could have seeded a bolus of bacteria into the patient’s bloodstream and precipitated an onset of sepsis. Tachycardia, tachypnea, fever, and hypotension are all hallmarks of severe sepsis. While this is beginning to look like a possibility, it raises the question of where and how this patient could have developed an abscess. A review of available case reports reveals numerous examples of ovarian and tubo-ovarian abscesses presenting years to over a decade following an initial hysterectomy.^2^–^6^ This patient was also uniquely at risk of an uncommon sexually transmitted infection (STI) that is on the rise within certain populations including men who have sex with men – lymphogranuloma venereum (LGV).^7^ This serovar of *Chlamydia trachomatis* is specifically noted to cause lower abdominal pain following rectal inoculation from retroperitoneal and pelvic lymph nodes that practitioners are often unable to palpate on exam. As this infection progresses from secondary to tertiary, patients can develop a perirectal abscess and many of the constitutional symptoms this patient experienced.

The final possible etiology of this patient’s symptoms is a potential drug reaction from the antibiotics administered a few hours before his decompensation. The only allergic reaction that could occur within that time frame would be a Type I, immunoglobulin E-mediated anaphylactic reaction. While anaphylaxis is commonly characterized by respiratory symptoms and hypotension, it does not typically cause fever and does not explain the patient’s chronic symptoms.^8^ The azithromycin and ceftriaxone the patient received are common and appropriate treatments for chlamydia and gonorrhea. But ceftriaxone can be used to treat other STIs as well, including chancroid and syphilis.

The treatment of syphilis, intentional or not, can also cause a different type of reaction. It is worth noting that secondary syphilis can cause many of the chronic symptoms we have been attempting to explain: fever, headaches, weight loss, myalgias, and fatigue. Interestingly, patients may exhibit a rash so faint that patients and providers do not notice it. When treated, many spirochetes like syphilis can cause a Jarisch-Herxheimer reaction.^9^ The symptoms of this reaction are contrasted to those of anaphylaxis in Table 2. Taking these symptoms into account, we believe such a reaction seems like a reasonable etiology of this patient’s striking presentation. This reaction is usually associated with penicillin, but has been previously reported after administration of ceftriaxone.^10^

Ultimately, we seek a single diagnosis that unifies what is seemingly a tale of two patients. The previous discussion has left us with two reasonable choices: an LGV abscess or a Jarisch-Herxheimer reaction. A computed tomography or nucleic acid amplification test might diagnose the former, while a rapid plasma regain (RPR) or venereal disease research laboratory test (VDRL) should confirm the latter. In deciding between these, I cannot help but think back to one of the key characters from Dickens’ *A Tale of Two Cities*, Sydney Carton. Mr. Carton ultimately met his demise in the turbulent chaos of Paris and, like many Europeans of the time, was suspected of having a specific venereal disease – syphilis! Hopefully this patient’s story concludes with a far more favorable outcome!

### Clinical Diagnosis

Jarisch-Herxheimer reaction following empiric treatment of syphilis.

## CASE OUTCOME (Dr. Emily Fleming)

Multiple labs were sent after the patient’s change, including blood cultures and lactate. Ultimately a positive RPR confirmed the diagnosis. Given the patient’s vital signs and overall appearance, he was kept overnight in an observation unit and treated supportively with IV fluids and antipyretics. He felt much better and was discharged to the following morning to follow up with his PCP.

Unfortunately, despite just having completed three weeks of IM penicillin, the patient returned to the ED with a severe headache, photophobia, and word-finding difficulties. He also reported that his generalized malaise and weakness had yet to fully resolve. He had a normal neurologic exam and head computed tomography and felt better after a “headache cocktail” of medications, but he presented once again a week later with severe headache and slurred speech and was ultimately diagnosed with neurosyphilis after a positive lumbar puncture. Infectious disease was consulted, a peripherally inserted central catheter line was placed, and the patient received two weeks of IV ceftriaxone in the treatment of neurosyphilis.

## RESIDENT DISCUSSION

The Jarisch-Herxheimer reaction is an acute febrile reaction that occurs within the first 24 hours of treatment. The pathophysiology is poorly understood but is thought to be due to a cytokine storm caused by the sudden release of bacterial products from injured or killed bacteria. This reaction has been reported in up to 30% of primary syphilis and up to 90% of secondary syphilis cases. Signs and symptoms include fever, myalgias, rigors, hypotension and rash.

Syphilis has been called the “great imitator” given its varied presentations. Primary syphilis is a local infection that will present as a painless ulcer, known as a chancre. It is often accompanied by moderate regional lymphadenopathy. This presents on average 21 days after infection. It is often missed by patients given its painless and self-resolving nature. Secondary syphilis occurs approximately 4–12 weeks after initial infection. At this point the infection is now systemic, and patients often endorse constitutional symptoms (myalgias, fatigue, weight loss). Rash is the most easily identifiable sign of secondary syphilis. It is classically diffuse, maculopapular, and is also found on the palms and soles (making it somewhat unique). Secondary syphilis can less commonly cause hepatitis, acute nephritis, and synovitis.^11^

Following early syphilis, there is often an asymptomatic period termed “latent syphilis.” Approximately 40% of patients with untreated early syphilis will develop tertiary syphilis anywhere from 1–30 years after initial infection. The manifestations of late syphilis are varied, but most commonly affect the cardiovascular and central nervous systems. While neurosyphilis is often thought of as a complication of tertiary syphilis, it can occur in any stage of the disease. Early neurosyphilis often presents with meningitis (fever, headache), uveitis (decreased visual acuity), or infectious arteritis (stroke-like symptoms). Late neurosyphilis classically presents as general paresis or tabes dorsalis. General paresis is associated with personality changes and progresses to severe dementia. Tabes dorsalis affects the posterior column of the spinal cord resulting in sensory ataxia.^12^

Lumbar puncture is recommended in patients with known syphilis and any neurologic symptoms, HIV, or an RPR > 1:32. Cerebrospinal fluid (CSF) studies will often show high white blood cell and protein counts. The VDRL test is a highly specific but poorly sensitive CSF study whereas the fluorescent treponemal antibody absorption test is highly sensitive but is often a send-out lab.^13^ Treatment of syphilis depends on the stage of the disease. Early syphilis is treated with a single IM dose of 2.4 million units penicillin G. Late syphilis, or syphilis of unknown duration, requires once weekly injections of 2.4 million units of penicillin G for three weeks. Neurosyphilis at any stage requires two weeks of IV penicillin or ceftriaxone.^14^

## FINAL DIAGNOSIS

Jarisch-Herxheimer reaction following treatment of neuro-syphilis.

## KEY TEACHING POINTS

1. LGV should be considered in high-risk groups such as men who have sex with men, and can lead to constitution symptoms and abscess formation if allowed to progress to tertiary stages.
2. The symptoms of anaphylaxis and a Jarisch-Herxheimer reaction can be clinically similar, but the latter typically occurs later (hours to days after drug administration), can cause hyperthermia, and should cause a worsening of the patient’s existing rash (in contrast to the hives that develop in anaphylaxis).
3. The classic rash associated with syphilis is made of diffusely spread asymptomatic maculopapular lesions that include the palms and soles, and may be so faint that it is overlooked by providers.

## Figures and Tables

**Table 1:** Laboratory results of a 37-year-old transgender male with sudden decompensation.

Lab test	Value	Units	Normal range
White blood cell count	9.5	K/mcL	4.5 – 11.0
Hemoglobin	11.7	g/dL	12.6 – 17.4
Hematocrit	34.0	%	37.0 – 50.0
Platelets	335	K/mcL	153 – 367
Sodium	133	mmol/L	136 – 145
Potassium	4.0	mmol/L	3.5 – 5.1
Chloride	99	mmol/L	98 – 107
Bicarbonate	21	mmol/L	21 – 30
Glucose	107	mg/dL	70 – 99
Creatinine	0.75	mg/dL	0.66 – 1.25
Blood urea nitrogen	11	mg/dL	7–20
Urine glucose	Negative		Negative
Urine specific gravity	1.015		1.002–1.030
Urine ketones	Trace		Negative
Urine nitrites	Negative		Negative
Urine leukocyte esterase	Trace		Negative
Urine WBC	0–5	/hpf	0–5
Urine RBC	0–5	/hpf	0–5
HIV	Nonreactive		Nonreactive
Wet prep	Negative		Negative
Fungal smear	Negative		Negative
Gonorrhea testing	Pending		Negative
Chlamydia testing	Pending		Negative

*K*, kilogram; *mcL*, microliter; *g*, grams; *dL*, deciliter; *mmol*, millimoles; *L*, liter; *mg*, milligrams; *WBC*, white blood cells; *RBC*, red blood cells; *hpf*, high-power field; *HIV*, human immunodeficiency virus.

**Table 2:** Similarities and differences between Jarisch-Herxheimer reaction and anaphylaxis.

	Jarisch-Herxheimer Reaction	Anaphylaxis
Onset	Varies by spirochete Occurs within hours to days of antibiotic administration	Within minutes to hours of stimulus
Symptoms	Tachycardia Hypotension Hyperventilation Worsening rash Fever Chills Rigors Headache Myalgias Shock (rarely)	Tachycardia Hypotension Bronchoconstriction Rash/Hives Angioedema Nausea/Vomiting Chest tightness Flushing Shock (possible) Death (possible)
